# Exploring the Role of Environmental Regulation and Fiscal Decentralization in Regional Energy Efficiency in the Context of Global Climate

**DOI:** 10.3390/ijerph192416577

**Published:** 2022-12-09

**Authors:** Qianqian Wu, Rong Wang

**Affiliations:** Business School, Nanjing Xiaozhuang University, No. 3601, Hongjing Avenue, Jiangning District, Nanjing 211171, China

**Keywords:** global climate change, environmental regulation, fiscal decentralization, energy efficiency, sustainable development

## Abstract

As an important factor of production, the use of energy will greatly improve production efficiency and is the material basis for China’s sustainable development. Environmental regulation plays an important role in energy efficiency (EE), while fiscal decentralization is an important institutional context for China’s economic development. In order to explore the role of environmental regulation and fiscal decentralization on EE, this paper measures the EE of 30 provincial regions in China from 2006 to 2020 by the super-efficient SBM method, and verifies the relationship between fiscal decentralization and environmental regulation on EE using a spatial econometric model, and conclusions as follows: (1) China’s overall energy efficiency averages 0.563, still at a low level, with the highest average efficiency value in the east and the lowest in the west. (2) An inverted U-shaped relationship exists between environmental regulation and EE, and its spatial spillover effect also shows an inverted U-shaped relationship. Fiscal decentralization promotes the regional EE, and its spatial effect also significantly positive. (3) When the level of environmental regulation increases, the EE level of the local and neighboring provinces will increase. The enhancement of fiscal decentralization system can promote local EE, but it has a negative effect on the energy efficiency level of neighboring provinces. Finally, based on the results of the empirical analysis, this paper proposes suggestions for improving regional EE, which are of great theoretical and practical value for improving climate change and achieving sustainable regional economic development in China.

## 1. Introduction

Energy is indispensable for human development. As long as human beings do not give up economic development, human demand and consumption of energy will continue to grow. An important consequence of energy use is the production of CO_2_, and 97% of CO_2_ in western industrial countries comes from emissions from energy use such as coal, fuel oil and gas [1], and in the 1990, Intergovernmental Panel on Climate Change’s Summary for Policy Makers stated that CO_2_ is the most significant greenhouse gas contributing to global warming. The Kyoto Protocol to the United Nations Framework Convention on Climate Change (UNFCCC) in 1997 clearly proposed a strategy for rationalizing energy use to prevent climate change, adjust the traditional mode of energy use, and improve EE [2]. Energy efficiency strategy will undoubtedly influence the future energy demand pattern and growth trend of the world.

EE refers to the reduction of energy inputs to provide equivalent energy services. The large amount of waste emitted during the inefficient use of energy has caused serious pollution to the environment and poses a threat to the quality development of China’s economy, social progress, and the welfare of its residents. In order to achieve sustainable development goals, the Chinese government is actively working to ameliorate the increasing resource shortages and environmental degradation while steadily developing the economy. The national 13th Five-Year Plan for economic and social development clearly states that green is a necessary condition for sustainable development and that the basic state policy of resource conservation and environmental protection must be adhered to [3]. Therefore, improving EE is of great significance to promote the synergistic development of “energy-economy-environment-society”. Improving EE and promoting sustainable economic development require a comprehensive examination of institutional factors and policy instruments, and fiscal decentralization and environmental regulation are undoubtedly very important aspects [4], especially since the formal implementation of the tax-sharing reform in China in 1994, the new system of fiscal and tax allocation between the central government and local governments has significantly influenced the level of local development, which in turn has had a profound impact on regional energy use, ecological environment, and social welfare. This has had a profound impact on regional EE, ecological environment, and social welfare. Therefore, it is important to include fiscal decentralization and environmental regulation in the analytical framework to study their effects on EE.

According to Porter’s hypothesis, appropriate environmental regulation can increase the investment of regulated enterprises in environmental protection, carrying out technological innovation, improving the competitiveness of enterprises, thus compensating for the losses caused by environmental regulation to a certain extent and improving energy efficiency. Another view based on the cost effect is that environmental regulations can increase the production costs of enterprises, add their burden, and inhibit technological progress, thus leading to a decrease in EE [5]. Finding a solution to the relationship between economic growth and energy and environment to improve EE is a great challenge for China’s sustainable economic development. Analyzing EE and its changes in the context of environmental regulation has a positive effect on achieving harmonious development of energy and environment. Therefore, the answers to questions such as what is the impact of environmental regulation on EE and is there any spatial effect, how can we achieve a “win-win” goal for environmental protection and EE improvement, will not only help evaluate the effectiveness of the existing environmental regulation policies, but also provide a theoretical basis and practical basis for the formulation of reasonable environmental regulation policies in the region.

But the Porter hypothesis ignores the “institutional” factors that are closely linked to it. Currently, China is in a relatively decentralized economic system and a relatively centralized financial system. The existence of fiscal decentralization makes lower-level governments face greater pressure for promotion while gaining power, i.e., to increase regional GDP, so that lower-level governments have to pursue high economic output at the cost of high inputs, high pollution, and high emissions [6,7,8,9,10]. Local government and industrial development are important factors affecting economic development and ecological environment, and are important carriers for giving full play to government functions and market functions, which can have an important impact on resource allocation efficiency and resource efficiency, and are also the key to reducing environmental pollution and building ecological civilization in China. However, many studies on environmental regulation and energy efficiency have neglected the influence of fiscal decentralization. Therefore, an in-depth study on the relationship between environmental regulation and fiscal decentralization on EE has important theoretical and application values for the coordinated development of resources, environment, and economy as well as the construction of ecological civilization in China.

Based on this, in the context of global climate change, this paper adopts the super-efficient SBM method to measure the EE of 30 provincial regions in China from 2006 to 2020, and uses a spatial econometric model to verify the relationship between fiscal decentralization and environmental regulation on EE. The main contributions of this paper are as follows: (1) using the super-efficient SBM method to calculate the energy efficiency values of each province and city, so as to make more accurate judgments on the energy efficiency level of each region and provide reference for regional EE evaluation. (2) The inclusion of fiscal decentralization and environmental regulation on energy efficiency in the same framework is a way to fill the existing research gaps and to expand and enrich the existing research perspectives. (3) The decomposition of spatial spillover effects can provide a policy basis for policy implementation between regions and is conducive to coordinated regional development.

## 2. Literature Review

### 2.1. Meaning and Measurement of EE

Generally speaking, efficiency includes two components: technical efficiency and allocative efficiency. The former is expressed as the approximation of a single production unit to the overall technical level of its industry in the process of production from the beginning to the end, and technical efficiency is more widely used compared to the two, so energy efficiency mostly implies the technical efficiency of energy [11]. Patterson [12] considers energy efficiency as the reduction of energy use while maintaining equal production and utilization of output of high value, this key point is to define energy inputs and outputs of high value in a reasonable way. Domestic scholars Ma and Yan [13] proposed that EE is the ratio of the optimal energy input to the actual energy input based on summarizing previous studies, and Jin [14] argued that two aspects need to be noted in grasping energy efficiency at the macro level: first, energy input needs to be reduced as much as possible in improving EE, and at the same time, output should not be decreased due to the reduction of input. The second is that the ultimate goal of improving EE is to reduce energy factors input, thus reducing production costs and forming a comparative advantage compared to the international level.

Regarding the measurement of EE, Mukherjee [15] analyzed manufacturing industry EE and the six most energy-intensive double-digit industries in the United States during 1970–2001 from the perspective of production theory using the DEA model; Lundgren et al. [16] estimated the EE of 14 manufacturing sectors in Sweden based on SFA model, and showed that there is still much room for improvement in the utilization of fuel and electricity in manufacturing industries; Moon and Min [17] used a two-stage DEA model to measure EE; Li and Li [18] built a new energy efficiency system based on the original based on the original indicator system, constructed a new total factor energy efficiency indicator system, i.e., separating energy inputs from other intermediate inputs, selecting the input factors of the production process as labor, capital, energy input factors, and other intermediate input factors except energy, and also including wastewater and waste gas as non-desired output values into the indicator system; Meng and Zou [19] used a combined PP and SFA model and constructed a static evaluation model to measure EE.

### 2.2. Fiscal Decentralization Impact on EE

Tiebout [20] suggested that consumers “vote with their feet” to choose the areas that can satisfy their preferences, which in turn motivates local governments to provide more and better public infrastructure services and improve resource allocation and energy efficiency. Therefore, fiscal decentralization can promote competition among local governments to provide a more suitable production and living environment for manufacturers and residents. Min et al. [21] found that fiscal decentralization significantly improved energy efficiency, while economic competition among local governments reduced energy efficiency. Ji and Dai [22] concluded that moderate fiscal decentralization contributes to the energy efficiency improvement effect of industrial structure upgrading. Qu et al. [23] found that fiscal decentralization has an inverted U-shaped relationship with CO_2_ emission reduction efficiency. Song et al. [24] based on a panel quantile regression model also found that a moderate level of fiscal decentralization is beneficial to EE improvement. Yang et al. [25] found that fiscal decentralization and political promotion can inhibit EE, but the joint incentive can improve energy efficiency. Zhou et al. [26] found that fiscal decentralization can improve EE, but the joint effect with local government competition is negative. These scholars’ studies suggest that there is a nonlinear effect between fiscal decentralization and EE.

### 2.3. Environmental Regulation Impact on EE

Environmental regulation impact on EE has been studied and empirically demonstrated by various scholars from different perspectives, and three main views have been formed. One is that the two have a linear relationship. There are three main cases of research results of linear influence, promoting support theory such as Ahmed et al. [27], Mandal [28], Hsu et al. [29], Sun and An [30]; hindering the support theory such as You and Gao [31] that the high cost effect inhibits energy efficiency improvement; moderate support theory such as Xu et al. [32] has shown that at this stage, the eastern part of China can maintain stability on the basis of the current regulation standard; the central part can consider moderate strengthening of environmental regulation; in the western part, five provinces such as Inner Mongolia need to improve the intensity of regulation, and Guizhou and other provinces need to be cautious about the strengthening of regulation, and can consider promoting the enhancement and improvement of double efficiency more by means of technology introduction and innovation. Second, there exists a non-linear relationship between them. Under nonlinearity, Zhang et al. [33] found that environmental regulation affects total factor EE as inverted U type, both in the current period and its lagged period value. Wang and Yang [34] reached the same conclusion. Third, there is heterogeneity in the results. For example, Chen and Zhang [35] found large differences of environmental regulation-related variables impact on EE in different regions; Dong and Han [36] also reached the same conclusion.

In summary, there is a well-developed research system on the meaning, evolution trend, measurement system, and method of environmental regulation in the relevant research results. A mature framework has been established for the definition, measurement, and application of energy efficiency. It provides a solid foundation for this paper. However, there are still some problems that need to be added and improved: (1) most of them do not include fiscal decentralization in the analysis framework. In fact, the competition among local governments exists in the areas of fiscal revenue and expenditure, performance evaluation, and economic development, which affects the way and strength of environmental regulation and the implementation of innovation strategies. However, many results do not take into account this important factor, which is not in line with the reality of our country and it will have some impact on the validity of the conclusions. (2) EE spatial spillover effects among provinces and cities are not examined. Many results ignore the spatial correlation, and the studies conducted are only isolated analyses that consider the influence of regional factors. In fact, there are complex competition and cooperation relationships between local governments in each region of China, and the geographical interaction is strong, there may be spatial spillover effects, which need to be further verified. Therefore, the study takes fiscal decentralization into account when exploring environmental regulation impact on EE. Meanwhile, the study will further investigate the spatial correlation between them due to the behavior of local governments to imitate the policies of neighboring regions.

## 3. Methods

### 3.1. Energy Efficiency Measurement Methods

For the measurement of energy efficiency, academics generally use the SFA method and DEA method. The advantages of the SFA method are that it takes into account the influence of random errors on the results, and must determine the form of the production function before studying the production process, the accuracy of the technical efficiency calculation, and the correlation between efficiency and the influencing factors. The disadvantage is that the method assumes that all manufacturers will not exceed the optimal “frontier” when producing, thus creating an inefficient fraction, and making efficiency comparisons impossible. The advantage of DEA is not necessary to set a specific functional form, so it is possible to avoid the structural bias caused by the wrongly set production function, as in the case of traditional accounting methods and SFA methods, so this paper uses the DEA analysis method used by most scholars to measure EE.

#### 3.1.1. Super Efficiency SBM

At present, the mainstream energy efficiency measurement methods include parametric and non-parametric analysis methods. The parametric method is stochastic frontier analysis (SFA), and many scholars have conducted in-depth research using this method. For example, Xiao and Xiao [37] measured the ecological governance performance based on SFA model; Zhang et al. [38] constructed green technology innovation efficiency index system using SFA model. However, an obvious drawback of this method is that it can only measure a single output, so this method is not suitable for measuring EE with multiple inputs and outputs. The non-parametric method is represented by data envelopment analysis (DEA), which is also the most widely applied method about measuring energy efficiency at present. Zhang and Sun [39] et al. used a three-stage DEA model to measure EE. In the existing research, current measurement of energy efficiency is dominated by non-parametric, and most scholars choose DEA-SBM model, but this model has the problem of multiple efficiency values of 1 and effective incomparability, while Super-SBM model effectively overcomes this problem, and the model can include non-directional non-desired outputs, so this paper adopts the Super-SBM model improved by Tone [40] to measure regional EE, where each province has *N* factor inputs $x=\left(x_{1},\cdots ,x_{N}\right)\in R_{N}^{+}$ and M desired outputs $y^{g}=\left(y_{1}^{g},\cdots ,y_{k}^{g}\right)\in R_{M}^{+}$, while generating *K* non-desired outputs $y^{b}=\left(y_{1}^{b},\cdots ,y_{k}^{b}\right)\in R_{K}^{+}$, in each period *t*(*t* = 1, …, *T*), the EE of the *i*-th regions is as follows:

(1) $$\mathrm{GE}=\min\frac{\frac{1}{m}\sum_{i=1}^{m}\frac{\bar{x_{i}}}{x_{i0}}}{\frac{1}{M+K}\left(\sum_{r=1}^{M}\frac{y_{r}^{-g}}{y_{i0}^{g}}+\sum_{r=1}^{K}\frac{y_{r}^{-b}}{y_{i0}^{g}}\right)}$$

(2) $$s.t.\bar{x}\geq \sum_{j=1,\neq 0}^{n}\lambda_{j}x_{j},y^{-g}\leq \sum_{j=1,\neq 0}^{n}\lambda_{j}y_{j}^{g},y^{-b}\leq \sum_{j=1}^{n}\lambda_{j}y_{j}^{b}\;$$

(3) $$\bar{x}\geq x_{0},y^{-g}\leq y_{0}^{g},y^{-b}\leq y_{0}^{b}$$

(4) $$\sum_{j=1,\neq 0}^{n}\lambda_{j}=1,y^{-g}\geq 0,\lambda \geq 0\;$$

#### 3.1.2. Input and Output Variables Selection

(1) Input variables

Labor, the number of employed persons is selected as the measure of labor force.

Capital, reflected by capital stock. The capital stock is estimated using the perpetual inventory method with the following formula.

(5) $$K_{i,t}=K_{i,t-1}\left(1-\delta \right)+I_{i,t}$$

where *K*, *I*, and *δ* are capital stock, current year investment, and depreciation rate, respectively, and *i* and *t* denote region and year. The current year investment is measured by total fixed asset formation and the depreciation rate is chosen as 9.6% with reference to Zhang et al. [41].

Energy, expressed in terms of total energy consumption, include crude oil, gasoline, kerosene, diesel, fuel oil, coal, coke, and natural gas energy sources, due to the different quantitative scale of the eight energy sources, in the calculation of the unified conversion of tons of standard coal.

(2) Output indicators

Desired output, the GDP is selected as the measure of desired output.

Non-desired output, CO_2_ emissions are selected to measure. The following formula is used to calculate carbon dioxide emissions.

(6) $${\mathrm{CO}}_{2}=\sum_{i=1}^{8}COQ_{i}\times COF_{i}\times \frac{44}{12}\;$$

where CO_2_ denotes carbon dioxide emissions $COQ_{i}$ is *i*-th energy source, $COF_{i}$ represents the carbon emission factor. The number of employees in some provinces and cities is missing, so time series analysis is used to complete the data.

### 3.2. Spatial Econometric Model

#### 3.2.1. Spatial Econometric Model Form

Spatial econometric models can study the spatial correlation of variables. Panel models can only simply analyze the interaction between variables and cannot verify the spatial correlation of variables. In particular, for gases, their spatial mobility generates temporal inertia on itself, i.e., variables in the previous period affect variables in the current period. Therefore, we need to determine the choice of spatial econometric models before conducting empirical spatial econometric analysis, and the specific spatial econometric models are introduced as follows.

(1) Spatial error model. This model is the spatial autocorrelation of variables on the error term, and its expression is:

(7) $$Y_{it}=\beta X_{it}+\mu_{i}+\lambda_{t}+\tau_{it},\tau_{it}=\gamma \overset{N}{\underset{j=1}{\sum }}W_{it}\delta_{it}+\varepsilon_{it\;}\;$$

β represents the coefficient of influence of *X* on *Y*, *τ* represents the spatial autocorrelation error term, and *γ* represents the spatial autocorrelation coefficient. $\lambda_{t}$ represents the time effect, *W* represents the spatial weight matrix, and ε represents the random disturbance term.

(2) Spatial lag model. The spatial correlation is the effect of the change in the dependent variable in the region due to the spatial lag term of the dependent variable, and the change in the dependent variable in the neighboring regions respectively. The SAR model is to study the degree of spillover effect, and its expression is:

(8) $$Y_{it}=\beta X_{it}+\mu_{i}+\lambda_{t}+\rho \overset{N}{\underset{j=1}{\sum }}W_{it}Y_{it}+\varepsilon_{it\;}$$

ρ is the effect of the change in the dependent variable in the neighboring region on the change in the dependent variable in the region, i.e., spatial autocorrelation.

(3) Spatial Durbin model. This model incorporates the “temporal inertia” and “spatial spillover effect” of the dependent variable, and its expression is as follows:

(9) $$Y_{it}=\beta X_{it}+\mu_{i}+\theta \overset{N}{\underset{j=1}{\sum }}W_{it}X_{it}+\varepsilon_{it}+\lambda_{t}+\rho \overset{N}{\underset{j=1}{\sum }}W_{it}Y_{it}\;$$

where *θ* is the effect of independent variable in the neighboring region on the dependent variable in the region.

Spatial autocorrelation test.

#### 3.2.2. Spatial Correlation Test

The most commonly used method in the existing literature is Moran’s I [42]. Greater than 0 indicates positive correlation, less than 0 indicates negative correlation. The calculation formula is:

(10) $$\mathrm{Moran}’s\;I=\frac{\sum_{i=1}^{n}\sum_{j=1}^{n}W_{ij}\left(X_{i}-\bar{X}\right)\left(X_{j}-\bar{X}\right)}{S^{2}\sum_{i=1}^{n}\sum_{j=1}^{n}W_{ij}}$$

In the formula, *n* represents number of regions; $X_{i},\;X_{j}$ is the attribute values of city *i* and city *j* respectively; $W_{ij}$ denotes the spatial weight matrix, and the economic distance weight matrix is chosen in this paper. 

#### 3.2.3. Spatial Weight Matrix Setting

The study drawing on the studies of Bivand and Wong [43] and Getis [44], the economic distance spatial weight matrix is constructed considering economic factors, and its spatial spillover effects are analyzed comparatively, respectively, economic distance also means the economic (GDP) gap between two regions, the formula is:

(11) $$W_{ij}=\{\;\begin{array}{c} 1/|\bar{y_{i}}-\bar{y_{j}}|\;i\neq j\\ \;0\;\;\;\;\;\;\;\;\;\;\;\;\;\;\;\;\;\;\;\;i=j \end{array}$$

#### 3.2.4. Spatial Spillover Effect Decomposition

In the spatial econometric model, the regression coefficients of the independent variables receive feed-back effects and thus become intricate. For this reason, the Lesage and Pace [45] proposed a specific impact effect decomposition method based on a generalized spatial model, combined with the study, the direct effect is *x* impact on the province’s own *y*, and the indirect effect is *x* impact on the neighboring province *y*, the total effect is their sum. Improvements to the original model are as follows.

(12) $$\left(I_{n}-\rho W\right)Y=\alpha l_{n}+\beta X+\theta WX+\varepsilon$$

(13) $$Y=\sum_{r=1}^{k}S_{r}\left(W\right)X_{r}+V\left(W\right)l_{n}\alpha +V\left(w\right)\varepsilon$$

(14) $$S_{r}\left(W\right)=V\left(W\right)\left(I_{n}\beta_{r}+W\theta_{r}\right)$$

(15) $$V\left(W\right)={\left(I_{n}-\rho W\right)}^{-1}=I_{n}+\rho W+\rho^{2}W^{2}+\rho^{3}W^{3}+\cdots$$

*r* is the number of independent variables, *X* is the *r*-th independent variable value, and *θ* is the *r*-th lagged variable coefficient of *W_r_*. Then, we have the following matrix relationship.

(16) $$\left(\begin{array}{c} Y_{1}\\ \begin{array}{c} Y_{2}\\ M\\ Y_{n} \end{array} \end{array}\right)=\overset{k}{\underset{r=1}{\sum }}\left(\begin{array}{c} S_{r}{\left(W\right)}_{11}\;S_{r}{\left(W\right)}_{12}\;\cdots \;S_{r}{\left(W\right)}_{1n}\;\\ \begin{array}{c} S_{r}{\left(W\right)}_{21}\;S_{r}{\left(W\right)}_{22}\;\cdots \;S_{r}{\left(W\right)}_{2n}\\ \cdots \;\ddots \;\vdots \\ S_{r}{\left(W\right)}_{n1}\;S_{r}{\left(W\right)}_{22}\;\cdots \;S_{r}{\left(W\right)}_{kn} \end{array} \end{array}\right)\left(\begin{array}{c} X_{1r}\\ \begin{array}{c} X_{2r}\\ \vdots \\ X_{kr} \end{array} \end{array}\right)+V\left(W\right)l_{n}\alpha +V\left(W\right)\varepsilon$$

(17) $$Y_{i}=\sum_{r=1}^{k}\left[S_{r}{\left(W\right)}_{i1}X_{1r}+S_{r}{\left(W\right)}_{i2}X_{2r}+\cdots S_{r}{\left(W\right)}_{ik}X_{kr}\right]+V\left(W\right)l_{n}\alpha +V\left(W\right)\varepsilon$$

(18) $$\frac{\partial Y_{i}}{\partial X_{jr}}=S_{r}{\left(W\right)}_{ij}$$

(19) $$\;\frac{\partial Y_{i}}{\partial X_{ir}}=S_{r}{\left(W\right)}_{ii}$$

$S_{r}{\left(W\right)}_{ij}$ is the indirect effect, while $S_{r}{\left(W\right)}_{ii}$ is the direct effect.

#### 3.2.5. Variable Selection

(1) Explained variables

Energy efficiency (EE): EE is measured by super-SBM in the study.

(2) Explanatory variables

Environmental regulation (ER): The choice of proxy variables for environmental regulation is mainly about availability and representativeness, refer to Dirckinck-Holmfeld [46]. Given that environmental regulation not only invests in pollution control, but also in environmental protection and improvement, the paper uses the ratio of total investment in environmental pollution control/total GDP of each region for measurement, where GDP is adjusted to 2006 constant prices to maintain the consistency of statistical caliber, and environmental regulation input is estimated by the perpetual inventory method and converted to 2006 constant prices.

Fiscal decentralization (FD). There are two ways to measure fiscal decentralization in the existing literature: one is to use the provincial government’s average or marginal share of the province’s budget revenue. The other, more common approach is to measure it from the perspective of local government fiscal revenues and expenditures. In this paper, borrowing from the method of He Jun et al. [47], fiscal decentralization is expressed as local fiscal expenditure per capita at this level/(local fiscal expenditure per capita at this level + central fiscal expenditure per capita at this level). The larger the value of the indicator, the higher the degree of fiscal decentralization and the greater the autonomy of provinces to utilize their own provincial finances.

(3) Control variables

Openness to the outside world (OPEN). The opening up to the outside world can promote EE through technological effects. The introduction of foreign direct investment will bring in capital as well as advanced production technology and management methods. On the one hand, foreign enterprises will choose to use energy-efficient technologies in order to reduce energy waste caused by excessive energy consumption, and the application of energy-efficient technologies can not only improve energy efficiency but also have certain benefits for solving environmental pollution problems; on the other hand, foreign direct investment can introduce foreign advanced technologies into the country, and the progress of technology can promote the improvement of energy efficiency in the country; in addition, opening up to the outside world through the structural effect, energy efficiency will be reduced. Developing countries with relatively backward economic development will choose to attract foreign enterprises by lowering environmental standards when pursuing economic growth, so it may bring more investment in high energy consumption and high pollution industries, which not only have low energy use efficiency but also cause serious damage to the environment [48]. In this paper, the ratio of the total import and export trade to the GDP of each province is used to express the level of foreign trade.

Industrial Structure (IS). Among the three industries, due to the inherent characteristics of industry, its energy efficiency tends to be lower than that of other industries. With the continuous development of industrialization process, the overall level of energy efficiency will be low. When the economic structure shifts to the service industry, it will improve energy efficiency to some extent because of its less energy consumption and lighter pollution emission. Changes in the internal structure of industries will have some impact on energy efficiency. In the case of industry, when it changes from resource-intensive industry to technology-intensive industry, energy efficiency will be improved when high pollution industry is converted to low pollution industry. Ma and Stern [49] shows that industrial structure has an important impact on EE. Because industry is more dependent on energy, the share of industrial value added in GDP is chosen to represent industrial structure in this paper.

Energy price (EP). Considering that the elasticity energy is related to the change of energy price, as the price of energy increases, on the one hand, it will raise the consciousness of enterprises to save, and on the other hand, enterprises will improve energy efficiency by developing new technologies and finding new energy sources for substitution. With reference to other scholars’ studies, energy prices are expressed as the purchase price index of fuel and power categories in the current year and converted to comparable prices.

Economic development (PGDP). Environmental Kuznets Curve: The hypothesis assumes a strong link between economic development and environmental damage and is expressed in terms of per capita gross regional product.

#### 3.2.6. Data Sources and Descriptive Statistics

The data are obtained from the website of the National Bureau of Statistics, the statistical yearbooks of various places and the monthly reports of China’s economic prosperity for the same period. Due to the lack of data for Tibet, Tibet is excluded and 30 provinces (cities and districts) are counted. Therefore, a total of 15 years of cross-sectional data are selected for this paper, with a total of 450 samples. The results of descriptive statistics are shown in Table 1 below.

## 4. Results

### 4.1. Energy Efficiency Measurement Results

**Figure 1:**
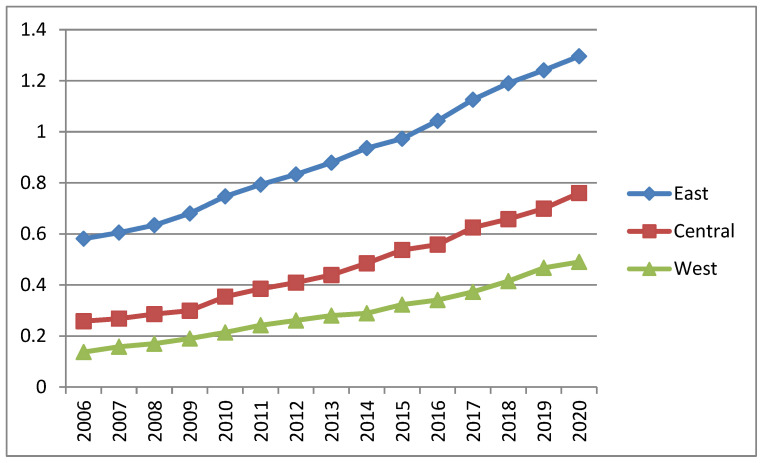
Trends of EE in the three regions.

The super-efficient SBM model (including non-consensual output) was used to measure the Chinese provincial regions EE from 2006 to 2020, and the specific efficiency values and their overall average values for each year, as shown in Table 2, and plotting the EE of the east, central, and west in Figure 1 to analyze the trend.

In Table 2, we can see that Chinese average EE fluctuates between 0.145 and 1.785 on an average, with significant differences in the horizontal comparison between provinces and municipalities, showing an undulating change overall. In the study period, among the 30 regions in China, the average EE of Beijing, Shanghai, and Guangdong is greater than 1, accounting for 10%; the average EE of Jiangsu and Zhejiang is between 0.8 and 1.0, accounting for 6.6%; the average EE of Anhui, Fujian, and Chongqing is between 0.6 and 0.8, while the rest is below 0.6. This indicates that during the period of 2006–2020, the EE of most regions generally did not reach the expected ideal state. From the ranking of regional averages of provinces in 2006–2020, the top five provinces all belong to developed provinces, which have been in the forefront of the country in terms of energy industry upgrading, energy structure transformation, and environmental protection.

Figure 1 reports the trends of EE in the three major regions of China, from which it can be seen that most of the eastern regions have higher EE indices, while most of the central and western regions have lower EE than the eastern level and the western region has the lowest. This coincides with the law of Chinese economic development. First, the industrial structure in the eastern is more rationalized and advanced, means that the industrial development is more inclined to clean industries. Second, the main support behind the more advanced industrial structure is the technology level, and the higher technology level is precisely conducive to improving EE, which can also explain the higher EE in the eastern region.

### 4.2. Spatial Econometric Analysis

#### 4.2.1. Multicollinearity Test

Before conducting the empirical evidence, we need to conduct the multicollinearity test on all the variable data before empirical evidence, and this paper uses the variance inflation factor (VIF) method to test the existence of multicollinearity. In Table 3, we can find that the values of VIF are all less than 10, and the average VIF is less than 5. In summary, it can be seen that there is no problem of multicollinearity.

#### 4.2.2. Panel Unit Root Test

The panel unit root test of variables can avoid the problem of “pseudo-regression”. There are four test methods, namely, LLC, HT, IPS, and Hadri LM test. Due to space limitation, after comparing the results of the above tests, we finally choose the LLC and Hadri LM tests to test the data stability (result in Table 4). In Table 4, the original hypothesis is rejected at the 5% significance level for all variables in the LLC test results, and the Hadri LM test results are rejected at the 1% significance level for all variables. In summary, all variables are stability, and there is no unit root in the panel data.

#### 4.2.3. Spatial Autocorrelation Test

Before empirical estimation, Moran’s I under economic distance weights is measured (result in Table 5). The Moran’s I values range from 0.34 to 0.41 throughout the study period, and they all pass the significance test, which indicates that China’s all-energy efficiency does not exhibit a completely random state, but there is a robust and significant spatial dependence.

#### 4.2.4. Spatial Econometric Model Selection

We need to perform the relevant tests before constructing the spatial econometric model to ensure the accuracy of model selection. The study uses the LM and LR methods for testing. Then, the Hausman test was used to determine whether the model used fixed effects or random effects, and the results of the above tests are shown in Table 6. The LM test rejected the original hypothesis, indicating that all three spatial econometric models could be used. The LR test rejected the original hypothesis that the SDM model would degenerate into the SLM model and SEM model, indicating that the selection of SDM was more accurate. The Hausman test rejects the original hypothesis of random effects, and therefore, the fixed effects model is chosen for the empirical analysis.

According to spatial Durbin model, the model is constructed as follows (5):

(20) $${GE}_{it}{=\alpha }_{0}{+\alpha }_{1}ER_{it}+\alpha_{2}{\left(ER_{it}\right)}^{2}{+\alpha }_{3}{FD}_{it}{+\alpha }_{4}{Col}_{it}{+\alpha }_{5}W_{ij}{*ER}_{it}{+\alpha }_{6}W_{ij}{*FD}_{it}\;{+\alpha }_{7}W_{ij}{*Col}_{it}{++\mu }_{i}{+v}_{t}{+\varepsilon }_{it}$$

${GE}_{it}$ is energy efficiency, $ER_{it}$ is environmental regulation, ${FD}_{it}$ is fiscal decentralization, and “Col” is the control variable, include openness to the outside world (OPEN), industrial structure (IS), energy price (EP), and economic development level (PGDP), respectively.

#### 4.2.5. Results of Spatial Econometric Analysis

In this paper, regression analysis was conducted using the relevant software designed by Elhorst (2010) [50], results shown in Table 7 below.

The positive coefficient of the primary term and negative coefficient of the secondary term of environmental regulation indicate that it has a significant inverted U-shaped relationship between environmental regulation and EE. When environmental regulation starts to increase, energy efficiency also increases; however, when it increases to a certain critical value, EE shows a decreasing trend. This is mainly because after the environmental regulations are implemented by the local government, the enterprises have to pay attention to the pollution problems caused by the government regulations, so they will not only work to reduce pollution emissions and use energy wisely, but also innovate production technology and plan the enterprise resource allocation in a timely manner, so that their energy efficiency can be improved. However, because the environmental problems are still serious and the goals set by the government have not been achieved, the government has accelerated the pace of regulation. In this context, some enterprises cannot bear the pressure exerted by the government and choose to merge and reorganize, which results in a continuous expansion of enterprise scale and an increase in industry intensity. At this time, some larger enterprises are able to update their equipment structure in time to improve energy utilization and thus reduce emissions. In addition, some enterprises often make promises to the government to reduce emissions when merging with other enterprises, thus passively improving environmental technology. Both of these approaches can lead to some improvement in energy efficiency. If the government chooses to continue to increase regulation, firms’ environmental costs will rapidly expand and their investment in purchasing efficient equipment and technology will increase, leaving them with less room for surplus production capital. On this basis, firms will become less energy efficient due to the heavy pressure exerted by the government.

The spatial spillover effect of environmental regulations also shows an inverted U-shaped relationship, means that the enhancement of environmental regulation promotes EE of other provinces, and when environmental regulation reaches a threshold, another increase in environmental regulation hinders regional energy efficiency in other provinces. On the one hand, neighboring provinces have convergence in policy formulation and implementation, and in the face of severe environmental governance pressure, neighboring provinces will choose more similar environmental governance policies, leading to the convergence of their environmental regulation levels. Due to the knowledge spillover effect, when environmental regulations in the region promote green innovation, the technology will flow rapidly among provinces, allowing other provinces to take advantage of the environmental governance.

The coefficients of fiscal decentralization are significantly positive, means that fiscal decentralization promotes the regional energy efficiency. The spatial effect of fiscal decentralization is also significantly positive, indicating that the spatial spillover effect cannot be ignored. So, the direct and indirect effects of the model must be decomposed and measured.

The regression coefficient of the level of openness is significantly positive, which may be due to the fact that the continuously open foreign trade improves the regional technological capability and factor productivity under the influence of technology spillover effect, further reducing energy consumption and pollutant emissions, and the spatial spillover effect is also significantly positive, indicating that the technological innovation brought by external openness has a spillover effect on the surrounding regions, which can significantly promote regional EE.

Industrial structure restricts EE because Chinese industry is still in the process of transformation and upgrading, and the intensive economic model has not been popularized nationwide, and many traditional industries are still in the “sloppy” growth mode, still consuming energy, capital, environment, and labor in exchange for economic growth, which has many disadvantages. Therefore, industrial structure impact on EE at this stage is negative, and its spatial spillover effect is also significantly negative, means that the secondary industry has a negative impact on EE in the surrounding regions.

Energy price impact on EE is positive, which indicates that the increase of energy price is beneficial to EE improvement in China, and there is also a significant spatial spillover effect.

Economic development all has positive effect on EE, means that with economic development, people may be more aware of energy conservation and have higher requirements for energy efficiency, which in turn will attach importance to energy conservation, reduce pollution emissions, and improve energy efficiency. Economic development of the neighboring cities has a significant negative effect on the energy efficiency of this city at the level of 10%, indicating that the neighboring cities may relax the requirements for energy development in the process of vigorously developing diversified industries, and the neighboring energy efficiency may decrease due to government competition.

#### 4.2.6. Analysis of Spatial Spillover Effect

The study uses the stata software to measure the direct effect, indirect effect, and total effect, results as shown in Table 8 below.

The direct effect of environmental regulation on provincial EE is 0.045, and it passes the 5% significance test, which means that with a 1% increase, the level of EE in this province will achieve an increase of 4.5%, i.e., environmental regulation can improve EE in the province. First, this confirms that the introduction of environmental regulation increases the additional cost of enterprises, but the increase in production costs is more of an “innovation compensation” effect on the EE of the province, which confirms that Porter’s hypothesis is valid in China. At the same time, stronger environmental regulation may force companies to reallocate resources in the long run, i.e., to directly invest more money and manpower in research and development of energy use technologies, thus reducing their long-term energy consumption and improving their emission levels. Second, the introduction of environmental regulation constraints creates a good screening mechanism for provincial enterprises, and in order to maintain their market advantage, some enterprises will improve and upgrade their production methods in order to enhance their overall production performance and drive up EE. At the same time, environmental regulation will eliminate some high energy consumption and high emissions enterprises due to industrial transformation and upgrading, and the increase in governance costs and industrial transformation and upgrading will also force enterprises to innovate in technology.

In terms of indirect effects, the regression coefficient is 0.095, i.e., environmental regulation in one province shows a positive effect on EE in its surrounding provinces, i.e., environmental regulation improves EE in neighboring provinces. Environmental regulation policies have public goods properties. When environmental regulation progresses in one province, it generates policy innovation spillover to neighboring provinces through channels such as meetings and exchanges, study visits and networks, so that neighboring provinces learn from advanced experiences and practices, resulting in inter-provincial policy diffusion. The governments of neighboring provinces integrate the learned experiences into the provincial environmental regulation management, and improve the provincial environmental regulation policies by improving laws and regulations, improving environmental compensation mechanisms, and innovating government environmental supervision and input methods, which eventually lead to provincial EE improvement. At the same time, the provinces benefit from the technological innovation results related to energy factor utilization caused by neighboring provinces environmental regulation through exchange and cooperation to improve their own energy efficiency, thus forming a positive effect of the environmental regulation of neighboring provinces on the technical capacity of energy factor utilization of the province, which eventually contributes to energy efficiency.

Among the direct effects, fiscal decentralization promotes energy efficiency of local enterprises, and a higher level of fiscal decentralization implies that local governments have a higher ability to regulate their own taxation, which is a practical motivation for local governments to cooperate with the central government to control environmental pollution, improve energy efficiency, and enhance social welfare. With the expansion of fiscal decentralization, local governments’ investment preferences for improving enterprises’ green innovation levels increase, and their investment preferences for public goods and services such as environmental pollution control increase accordingly, and they formulate industrial policies with comparative advantages according to their own local characteristics. Therefore, the expansion of fiscal decentralization enhances the ability of local governments to provide public goods, and improving regional energy efficiency. Its indirect effects are negative, indicating that with regard to the performance competition among regional governments, government officials in the neighboring regions choose to carry out undesirable competition with the relaxation of environmental governance as a bargaining chip, and this practice leads to an ineffective EE improvement in the neighboring regions.

## 5. Conclusions and Implications

### 5.1. Conclusions

In the face of rapid economic development and accelerating industrialization, excessive energy consumption and ecological degradation have posed serious threats to sustainable economic development and global climate change. At present, there is a consensus in political and academic circles to improve energy efficiency and achieve low-carbon economic development. Environmental regulation, as an important factor that cannot be ignored for EE improvement, can influence EE through both positive and negative aspects. Considering the influence of the institutional context of fiscal decentralization, it must be included in this research system when studying the relationship between environmental regulation and EE. Fiscal decentralization is an important institutional feature of China’s economic development, and the process of environmental regulation and EE is closely related to it. The specific findings of this paper are as follows: 

(1) The average value of EE in China as a whole fluctuates between 0.145 and 1.785, with three regions of Beijing, Shanghai, and Guangdong greater than 1, accounting for: 10%; Jiangsu and Zhejiang average efficiency values are between 0.8 and 1.0, accounting for 6.6%; Anhui, Fujian, and Chongqing average efficiency values are between 0.6 and 0.8, and the rest are below 0.6. Most of the regions have higher energy efficiency index in the east, while most of the lower energy efficiency exists in the other two big regions.

(2) The positive coefficient of the primary term and negative coefficient of the secondary term of environmental regulation indicate that it has a significant inverted U-shaped relationship between environmental regulation and EE. The spatial spillover effect of environmental regulations also shows an inverted U-shaped relationship, which means that the enhancement of environmental regulation promotes other provinces EE, and when environmental regulation reaches a threshold, another increase in environmental regulation hinders regional energy efficiency in other provinces. 

The coefficients of fiscal decentralization are significantly positive, which means that fiscal decentralization promotes the regional energy efficiency. The spatial effect of fiscal decentralization is also significantly positive, indicating that the spatial spillover effect cannot be ignored. So, the direct and indirect effects of the model must be decomposed and measured.

(3) Environmental regulation direct effect on provincial EE is positive, environmental regulation can improve EE in the province, which confirms that Porter’s hypothesis is valid in China. In terms of indirect effects, environmental regulation in one province shows a positive effect on EE in its surrounding provinces, i.e., environmental regulation improves EE in neighboring provinces. 

Fiscal decentralization direct effect is positive in promoting the energy efficiency of local enterprises, but its indirect effects are negative, in the context of the existence of performance competition among regional governments, government officials in neighboring regions choose to carry out undesirable competition with relaxed environmental governance as a bargaining chip, and this practice leads to ineffective improvement of EE in the neighboring regions.

(4) For control variables, openness and energy price have positive direct and indirect effects, and industrial structure has negative direct and indirect effects, but economic development has positive direct and negative indirect effects due to government competition leading to a decrease in EE in the periphery.

### 5.2. Recommendations

Through the study of the impact of environmental regulation on energy efficiency under fiscal decentralization, the main conclusion has obvious policy implications, which is to give full play to the benefits of fiscal decentralization and avoid the negative effects of fiscal decentralization as much as possible. On the one hand, moderate fiscal decentralization plays an important role in improving energy efficiency; on the other hand, environmental regulation also has a direct role in promoting energy efficiency. Therefore, it is extremely important to make full use of the advantages of fiscal decentralization in order to give full play to the role of environmental regulation in improving energy efficiency in the context of good fiscal decentralization. Based on this, this paper proposes the following policy recommendations:

(1) To establish an efficient energy market, the most powerful price instrument in the market should not be ignored. Energy prices should reflect their true costs and government subsidies should be eliminated. Because subsidized energy products are cheaper, consumers will not choose energy products generated by energy efficiency projects, and investors will lose interest in energy efficiency utilization projects. Subsidized energy prices, therefore, actually encourage inefficient energy use and breed inappropriate energy use that harms the environment. A reasonable energy price should also include all marginal costs—the costs of overcoming environmental “external diseconomies” caused by energy consumption, including environmental and social marginal costs. In the context of energy use related to climate change, the environmental marginal cost of energy is specifically the “value of carbon”. An energy price that reflects all marginal costs will greatly improve EE.
(2) Define the characteristics of provincial industrial development and consolidate differentiated environmental policies. For different provinces and regions, industrial development characteristics and environmental protection standards differ due to many factors such as geographical location, openness, environmental carrying capacity, and resource endowment level. Under the premise of the overall national policy, local environmental regulation policies should be reasonably formulated. For example, the industrial economy in western provinces is relatively backward, with a high proportion of productive industries and a fragile ecological foundation, so it is necessary to enforce environmental regulation, strengthen the flow of information, and promote the transformation and upgrading of provincial industries. While the eastern provinces are now basically at the middle and end of industrial transformation and upgrading, the environmental regulations under the new industrialization should be changed and the standard of environmental requirements should be improved.
(3) Cross-regional environmental matters should be managed by the central government, and the government should take the lead and clarify the governance responsibilities of each region concerned. This is also due to the consideration of spatial autocorrelation of energy efficiency. In cross-regional environmental protection and pollution control activities, the control of pollution in one place will have obvious positive externalities for neighboring areas; the control of pollution in one place will also have obvious negative externalities for neighboring areas. Without the unified management and command of the central government, local governments may not provide sufficient financial spending efforts for such matters.
(4) Reasonable division of financial and administrative powers should exist between the central and local governments and enhancement of local governments’ financial power. This will therefore result in appropriately enhancing the financial power of local governments, improving local government revenue, and cultivating stable financial resources for local governments to promote EE, redistributing the tax revenue attributed to the central and local governments according to the responsibility of fiscal expenditure, and adjusting the distribution ratio of shared tax to increase the financial revenue of local governments. Meanwhile, the central government’s authority and expenditure responsibilities are appropriately increased, and the central government’s expenditure on public goods with strong positive externalities is strengthened.

### 5.3. Limitations and Prospects

(1) To investigate the effect of different types of environmental regulations on energy efficiency, such as classifying environmental regulations into command environmental regulations and incentive environmental regulations according to their types to study their effects.
(2) The optimal choice of environmental regulation. There is no definite conclusion on the optimal method to measure the intensity of environmental regulation. In this paper, we use the ratio of total investment in environmental pollution control/total GDP of each region for the reasonableness and availability of data, which is reasonable, but due to the different situations of each region and the continuous innovation of environmental regulation tools, we still need to combine the actual situation and conduct in-depth research to find a more suitable measurement method. Future research can explore the use of the integrated index method, using the emission of industrial wastewater, industrial waste gas, and industrial solid waste—three kinds of waste from the perspective of environmental regulation performance to build a comprehensive measurement system of China’s industrial environmental regulation intensity.
(3) In the study of the factors influencing energy efficiency, this paper has considered more macroeconomic-level influencing factors and has not analyzed them at a smaller micro level, such as the governance structure and property rights arrangement of enterprises, and future research can explore the micro-level perspective and expect to obtain richer conclusions.

## Figures and Tables

**Table 1:** Descriptive statistics of variables.

Variable	Sample Size	Mean	Standard Deviation	Minimum	Maxim
GE	450	0.563	0.441	0.061	2.421
ER	450	0.102	0.095	0.012	0.124
FD	450	0.887	0.456	0.456	2.127
OPEN	450	0.197	0.159	0.138	0.234
IS	450	0.587	0.441	0.347	0.772
EP	450	0.765	0.877	0.529	2.167
PGDP	450	5.236	4.432	1.56	7.183

**Table 2:** EE measurement results.

	Region	2006	2007	2008	2009	2010	2011	2012	2013	2014	2015	2016	2017	2018	2019	2020	Mean
Eastern	Beijing	1.097	1.141	1.242	1.349	1.476	1.577	1.682	1.787	1.879	1.946	2.224	2.256	2.343	2.355	2.421	1.785
	Tianjin	0.312	0.322	0.341	0.368	0.405	0.428	0.457	0.517	0.543	0.556	0.661	0.778	0.822	0.872	0.889	0.551
	Hebei	0.278	0.234	0.371	0.422	0.438	0.472	0.519	0.534	0.613	0.624	0.635	0.678	0.727	0.785	0.819	0.543
	Liaoning	0.243	0.256	0.329	0.333	0.448	0.463	0.478	0.485	0.491	0.508	0.519	0.532	0.641	0.725	0.741	0.479
	Shanghai	1.003	1.015	1.026	1.117	1.179	1.286	1.345	1.464	1.513	1.526	1.636	1.778	1.846	1.917	2.184	1.456
	Jiangsu	0.692	0.721	0.738	0.751	0.768	0.785	0.793	0.821	0.889	0.943	0.986	1.178	1.228	1.315	1.379	0.932
	Zhejiang	0.698	0.737	0.756	0.797	0.805	0.816	0.829	0.838	0.941	0.959	0.965	1.175	1.271	1.343	1.362	0.953
	Fujian	0.518	0.626	0.518	0.626	0.647	0.722	0.745	0.781	0.812	0.861	0.885	0.911	0.922	0.943	0.961	0.765
	Shandong	0.342	0.361	0.372	0.381	0.486	0.492	0.523	0.535	0.543	0.657	0.672	0.684	0.789	0.796	0.834	0.564
	Guangdong	0.894	0.918	0.943	0.968	1.164	1.256	1.335	1.441	1.589	1.612	1.746	1.852	1.877	1.927	1.978	1.433
	Hainan	0.311	0.328	0.341	0.368	0.405	0.428	0.457	0.466	0.483	0.516	0.541	0.568	0.622	0.672	0.689	0.480
Central	Shanxi	0.219	0.227	0.249	0.257	0.324	0.337	0.422	0.453	0.459	0.465	0.471	0.542	0.594	0.637	0.672	0.422
	Jilin	0.225	0.237	0.245	0.267	0.343	0.361	0.411	0.426	0.433	0.506	0.558	0.562	0.574	0.629	0.681	0.431
	Heilongjiang	0.221	0.233	0.241	0.253	0.298	0.345	0.315	0.426	0.436	0.543	0.533	0.641	0.682	0.754	0.765	0.446
	Anhui	0.315	0.327	0.385	0.397	0.462	0.478	0.543	0.571	0.673	0.732	0.764	0.861	0.918	0.957	0.969	0.623
	Jiangxi	0.223	0.235	0.243	0.255	0.337	0.342	0.346	0.352	0.454	0.531	0.578	0.595	0.611	0.627	0.729	0.431
	Henan	0.218	0.221	0.238	0.261	0.331	0.343	0.348	0.367	0.459	0.464	0.466	0.578	0.629	0.681	0.735	0.423
	Hubei	0.313	0.332	0.343	0.352	0.368	0.423	0.434	0.441	0.448	0.532	0.562	0.616	0.631	0.659	0.763	0.481
	Hunan	0.326	0.333	0.346	0.353	0.367	0.448	0.454	0.477	0.515	0.522	0.534	0.603	0.625	0.648	0.768	0.488
Western	Neimenggu	0.121	0.129	0.131	0.149	0.163	0.221	0.236	0.243	0.257	0.262	0.277	0.384	0.393	0.421	0.476	0.258
	Guangxi	0.107	0.118	0.137	0.148	0.153	0.221	0.235	0.253	0.242	0.289	0.302	0.321	0.333	0.452	0.464	0.252
	Chongqing	0.321	0.347	0.361	0.447	0.541	0.588	0.612	0.637	0.659	0.693	0.722	0.748	0.782	0.811	0.842	0.607
	Sichuan	0.272	0.273	0.292	0.313	0.325	0.336	0.384	0.393	0.395	0.403	0.411	0.416	0.422	0.432	0.441	0.367
	Guizhou	0.115	0.119	0.135	0.149	0.186	0.227	0.233	0.236	0.238	0.242	0.244	0.348	0.351	0.459	0.465	0.250
	Yunnan	0.121	0.222	0.231	0.252	0.256	0.243	0.245	0.255	0.272	0.386	0.391	0.405	0.412	0.522	0.561	0.318
	Shaanxi	0.118	0.123	0.138	0.143	0.213	0.239	0.254	0.282	0.315	0.413	0.422	0.437	0.513	0.542	0.556	0.314
	Gansu	0.087	0.099	0.107	0.119	0.121	0.139	0.166	0.237	0.225	0.232	0.248	0.256	0.359	0.378	0.326	0.207
	Qinghai	0.066	0.071	0.086	0.091	0.098	0.116	0.128	0.139	0.122	0.142	0.161	0.172	0.212	0.233	0.335	0.145
	Ningxia	0.061	0.072	0.081	0.092	0.083	0.105	0.119	0.121	0.136	0.137	0.159	0.174	0.258	0.351	0.369	0.155
	Xinjiang	0.122	0.163	0.172	0.183	0.216	0.229	0.254	0.282	0.315	0.352	0.412	0.437	0.528	0.534	0.556	0.317
National mean		0.332	0.351	0.371	0.399	0.447	0.482	0.510	0.542	0.578	0.618	0.656	0.716	0.764	0.813	0.858	0.563

**Table 3:** Multicollinearity test results.

Variable	VIF	1/VIF
ER	1.42	0.70
FD	2.35	0.43
OPEN	1.58	0.63
IS	2.59	0.39
EP	4.13	0.24
PGDP	3.25	0.31
Mean VIF	2.55	

**Table 4:** Unit root test.

Variable	LLC Test	*p* Value	LM Test	*p* Value
*GE*	−4.657	0.000	33.892	0.000
ER	−3.453	0.022	40.235	0.000
FD	−9.541	0.001	23.806	0.000
OPEN	−7.228	0.000	17.034	0.000
IS	−11.346	0.001	18.362	0.000
EP	−9.146	0.000	22.557	0.000
PGDP	−7.117	0.000	35.913	0.000

**Table 5:** Spatial autocorrelation test results.

Year	GE		Year	GE	
	Moran	*p* Value		Moran	*p* Value
2006	0.3452	0.0000	2014	0.3885	0.0000
2007	0.3644	0.0001	2015	0.3854	0.0002
2008	0.3564	0.0000	2016	0.3902	0.0001
2009	0.3786	0.0002	2017	0.3916	0.0000
2010	0.3665	0.0003	2018	0.3932	0.0000
2011	0.3457	0.0000	2019	0.3947	0.0000
2012	0.3579	0.0002	2020	0.4022	0.0000
2013	0.3763	0.0000			

**Table 6:** Spatial econometric model test results.

Year	GE	
	Moran	*p* Value
LM-lag	11.283	0.0000
R-LM-lag	9.728	0.0000
LM-error	7.881	0.0001
R-LM-error	6.021	0.0000
LR test for SLM	26.034	0.0002
LR test for SEM	26.773	0.0000
Hausman test	47.364	0.0000

**Table 7:** Spatial panel Durbin model regression results.

Variables	Regression Coefficient	Variables	Regression Coefficient
ER	0.135 ***	W*ER	0.157 ***
ER2	−0.224 ***	W*ER2	−0.216 ***
FD	0.278 ***	W*FD	0.318 ***
OPEN	0.027 ***	W*OPEN	0.121 ***
IS	−0.052 *	W*IS	−0.168 **
EP	0.012 ***	W*EP	0.053 ***
PGDP	0.1561 **	W*PGDP	−0.1007 *
W*dep.var	0.1823 ***		
R-squared	0.9912		
log-likelihood	418.348		

Note: * means *p* < 0.1, ** means *p* < 0.05, *** means *p* < 0.01.

**Table 8:** Decomposition results of spatial spillover effects.

Variables	Direct Effect	Indirect Effect	Total Effect
ER	0.045 **	0.095 ***	0.1400 ***
FD	0.156 ***	−0.0528 ***	0.1032 ***
OPEN	0.0454 ***	0.0329	0.0783 ***
IS	−0.1127 ***	−0.1388 ***	−0.2515 ***
EP	0.0934 ***	0.1276	0.2210 ***
PGDP	0.2418	−0.0672	0.1746 **

Note: ** means *p* < 0.05, *** means *p* < 0.01.

## Data Availability

The data used to support the findings of this study are available from the corresponding author upon request.
